# Metabolomics of Synovial Fluid and Infrapatellar Fat Pad in Patients with Osteoarthritis or Rheumatoid Arthritis

**DOI:** 10.1007/s10753-021-01604-x

**Published:** 2022-01-18

**Authors:** Petteri Nieminen, Wilhelmiina Hämäläinen, Juha Savinainen, Marko Lehtonen, Saara Lehtiniemi, Juho Rinta-Paavola, Petri Lehenkari, Tommi Kääriäinen, Antti Joukainen, Heikki Kröger, Tommi Paakkonen, Anne-Mari Mustonen

**Affiliations:** 1 Institute of Biomedicine, School of Medicine, Faculty of Health Sciences, University of Eastern Finland, P.O. Box 1627, 70211 Kuopio, Finland; 2 Department of Computer Science, Aalto University, P.O. Box 15400, 00076 Aalto, Finland; 3 School of Pharmacy/Pharmaceutical Chemistry, Faculty of Health Sciences, University of Eastern Finland, P.O. Box 1627, 70211 Kuopio, Finland; 4 Cancer and Translational Medicine Research Unit, Faculty of Medicine, University of Oulu, P.O. Box 5000, 90014 Oulu, Finland; 5 Department of Surgery and Medical Research Center, Oulu University Hospital, P.O. Box 21, 90029 OYS Oulu, Finland; 6 Pohjola Hospital, Leväsentie 1, 70700 Kuopio, Finland; 7 Department of Orthopaedics, Traumatology and Hand Surgery, Kuopio University Hospital, P.O. Box 100, 70290 Kuopio, Finland; 8 Department of Environmental and Biological Sciences, Faculty of Science and Forestry, University of Eastern Finland, P.O. Box 111, 80101 Joensuu, Finland

**Keywords:** high-resolution mass spectrometry, joint disease, metabolomics, osteoarthritis, rheumatoid arthritis, synovial fluid.

## Abstract

Osteoarthritis (OA) and autoimmune-driven rheumatoid arthritis (RA) are inflammatory joint diseases with complex and insufficiently understood pathogeneses. Our objective was to characterize the metabolic fingerprints of synovial fluid (SF) and its adjacent infrapatellar fat pad (IFP) obtained during the same surgical operation from OA and RA knees. Non-targeted metabolite profiling was performed for 5 non-inflammatory trauma controls, 10 primary OA (pOA) patients, and 10 seropositive RA patients with high-resolution mass spectrometry-based techniques, and metabolites were matched with known metabolite identities. Groupwise differences in metabolic features were analyzed with the univariate Welch’s *t*-test and the multivariate linear discriminant analysis (LDA) and principal component analysis (PCA). Significant discrimination of metabolite profiles was discovered by LDA for both SF and IFP and by PCA for SF based on diagnosis. In addition to a few drug-derived substances, there were 16 and 13 identified metabolites with significant differences between the diagnoses in SF and IFP, respectively. The pathways downregulated in RA included androgen, bile acid, amino acid, and histamine metabolism, and those upregulated included biotin metabolism in pOA and purine metabolism in RA and pOA. The RA-induced downregulation of androgen and bile acid metabolism was observed for both SF and IFP. The levels of 11 lipid metabolites, mostly glycerophospholipids and fatty acid amides, were also altered by these inflammatory conditions. The identified metabolic pathways could be utilized in the future to deepen our understanding of the pathogeneses of OA and RA and to develop not only biomarkers for their early diagnosis but also therapeutic targets.

**Supplementary Information:**

The online version contains supplementary material available at 10.1007/s10753-021-01604-x.

## INTRODUCTION

Osteoarthritis (OA) and autoimmune-driven rheumatoid arthritis (RA) are degenerative joint diseases that cause significant morbidity and a high demand for health services, including pain medication, rehabilitation, and surgery [1, 2]. While both OA and RA are considered inflammatory diseases, RA synovial tissue is characterized by higher immune cell infiltration and cytokine expression [3]. In addition to damage to cartilage and subchondral bone [4, 5], both conditions induce alterations in the composition of synovial fluid (SF) [6, 7]. The histological and biochemical profiles and secretory activity of the infrapatellar fat pad (IFP) of the knee can also be susceptible to joint diseases [7–9]. Pain and joint dysfunction are the principal symptoms of OA and RA. In order to discover new targets for disease prevention, early diagnosis, and pain management, it is crucial to assess the biochemical correlates and metabolic pathways related to subjective pain and disease prognosis. Here, the chemical milieu of SF would be an attractive target for research, as radiology has proven to be an imprecise predictor for pain symptoms [10], and it cannot readily discern the earliest stages of OA. Early diagnosis of joint diseases would be of pivotal importance, as substantial and irreversible joint pathology is usually present by the time of diagnosis.

Previous literature on the metabolomic manifestations of OA and RA indicates that they can be associated with dysregulation of several metabolic pathways. For instance, amino acid (AA) metabolism, fatty acid (FA) biosynthesis, glycero-PL (phospholipid) metabolism, oxylipin pathways, tricarboxylic acid cycle, and steroid hormone biosynthesis can be affected by joint diseases [11–14]. Regarding lipidology, the compositions of SF and IFP show complex effects of OA and RA [7, 15]. RA was manifested as increased concentrations of cholesterol, lipoproteins, and apolipoproteins in SF [16], and both OA and RA displayed higher levels of glycero-PLs and sphingolipids [17, 18]. In addition, the proportions of pro-inflammatory n-6 polyunsaturated FAs (PUFAs) decreased and the percentage of total monounsaturated FAs increased in both knee OA and RA SFs and, in knee OA, there was also a reduction in the proportions of 22:6n-3, a long-chain anti-inflammatory n-3 PUFA [7]. RA in the shoulder joint was characterized with elevated percentages of 20:4n-6 and 22:6n-3, and with reduced proportions of 18:1n-9 [15]. In IFP, RA patients showed decreased proportions of 20:4n-6, 22:6n-3, and total n-6 PUFAs compared to those with OA [7]. These findings indicate inflammatory phenomena but, at the same time, there are counteracting and resolving processes that could limit inflammation and eventually reduce both pain and cartilage degeneration.

Existing studies on OA and RA metabolomics mostly focus on SF, serum, and urine but, to the best of our knowledge, there are no simultaneous analyses of SF and IFP metabolites in these diseases. As it has been previously suggested that IFP could be a potential source of inflammatory or protective agents [11, 19], parallel analyses of SF and IFP can offer new ways to understand the sources of inflammatory molecules and metabolic pathways leading to the OA and RA metabolomic fingerprints in SF. Our aim was to conduct a metabolomic study including two diagnoses (primary OA [pOA] and RA) and two tissues (SF and, for the first time, also the adjacent IFP). This would be the starting point to translational studies to examine the influence of the fat pad on joint diseases, to assess potential biomarkers for early-stage OA and RA, and to screen for targets for therapeutic intervention. It was hypothesized that we would (i) detect significant differences between the SF metabolomes of the non-inflammatory control (C) patients and the inflammatory pOA and RA patients, (ii) observe similarities in the metabolomic signatures of arthritic SF and IFP, and (iii) detect tissue- or diagnosis-related differences between SF and IFP, all of which would be adding to our understanding of the characteristics of these diseases.

## MATERIALS AND METHODS

### Patients, Sampling, and Sample Preparation

The SF samples were obtained from the knee joints of patients undergoing arthroscopy for non-inflammatory conditions or trauma with no evidence of OA/RA (*n* = 2 men, 3 women), or during total joint replacement surgery for end-stage pOA (*n* = 2 men, 8 women) or end-stage seropositive RA (*n* = 3 men, 7 women) at the Oulu and Kuopio University Hospitals (Table 1). The IFP samples were collected from the same pOA and RA patients during the removal of periarticular tissues. All samples were stored at −70 °C until analyzed. The study was approved by the Ethical Committees of the respective hospitals (Oulu: decision #29/2011, amendment 2/24/2014; Kuopio: decision #79//2013, #73/2016) in accordance with the Helsinki Declaration. All patients had reached the age of majority (18 years) and were treated for pre-existing indications (Additional file 1), and they had signed consent forms to donate their SF and/or IFP samples. General demographic data collected were as follows: gender, age, body mass, height, body mass index (BMI), type of invasive procedure, operative diagnosis, and medication. Groupwise differences in age, body mass, and BMI were analyzed with the Kruskal–Wallis one-way analysis of variance (ANOVA), and sex ratios were compared with the Fisher’s exact test (IBM SPSS *v*25 software, IBM, Armonk, NY, USA).

**Table 1:** General Characteristics of the Sampled Knee Surgery Patients (Mean ± SE)

**Group**	**Control**	**pOA**	**RA**	***p***
Gender	2M, 3F	2M, 8F	3M, 7F	0.850
Age	34.4 ± 4.25^A^	66.7 ± 2.72^B^	71.9 ± 2.66^B^	0.002
Body weight	77.9 ± 7.07	85.4 ± 5.17	67.2 ± 5.97	0.103
BMI	27.0 ± 2.35^AB^	31.8 ± 1.98^B^	24.9 ± 1.50^A^	0.030

*pOA* primary osteoarthritis, *RA* rheumatoid arthritis, *M* male, *F* female, *BMI* body mass index; sex ratios were tested with the Fisher’s exact test; means with dissimilar superscript letters indicate significant differences between diagnoses within a row (Kruskal–Wallis ANOVA)

The SF samples consisted of 5 C, 10 pOA, and 9 RA patients and the IFP samples included 10 pOA and 10 RA patients. The samples were blinded, randomized, and thawed on ice. SF (25 μl) was mixed with 100 μl of ice-cold MeOH, shaken for 5 min (Multi Reax, Heidolph Instruments, Schwabach, Germany), and centrifuged at 2300 × *g* for 10 min at + 4 °C. The supernatant was transferred to test tubes and evaporated to dryness under nitrogen atmosphere, and the residue was reconstituted in 100 µl of 1:1 solution of ACN and H_2_O (v/v). The sample was allowed to dissolve for 10 min. The IFP samples were weighed (19–49 mg wet wt), placed in 100% MeOH at 10 mg/100 µl, and homogenized in a microtube (2 ml, reinforced with 1.4 mm ceramic beads) with the Bead Ruptor 24 Elite (Omni International, Kennesaw, GA, USA) at + 2 °C with a 30-s cycle. The samples were subsequently centrifuged at 1900 × *g* for 5 min at + 4 °C, and the clear supernatant was used for analysis. A small portion, approximately 10 µl of fat tissue and SF, was pooled and used as quality controls injected at the beginning and end of the worklist to equilibrate the analytical platform and to produce ion scanning.

### Instrumentation

The samples were transferred to the non-targeted metabolite profiling analysis carried out at the LC–MS metabolomics center (Biocenter Kuopio, University of Eastern Finland). The analysis was performed using the ultra-high-performance liquid chromatography quadrupole-time-of-flight mass spectrometry (UHPLC-qTOF-MS) system (Agilent Technologies, Waldbronn, Karlsruhe, Germany), which consisted of a 1290 LC system, a Jetstream electrospray ionization (ESI) source, and a 6540 ultra-high-definition accurate-mass qTOF-MS. To meet the wide diversity of molecular components, all samples were analyzed with 2 different chromatographic techniques: reversed phase (RP) and hydrophilic interaction chromatography (HILIC). In addition, data were acquired in both ionization polarities: ESI positive (ESI +) and ESI negative (ESI −). The temperature of the sample tray was maintained at + 10 °C. The data acquisition software was the MassHunter Acquisition B.04.00 (Agilent Technologies).

In the RP method, 2 μl of the sample solution was injected onto a column (Zorbax Eclipse XDB-C8, 2.1 × 100 mm, 1.8 µm, Agilent Technologies, Palo Alto, CA, USA) kept at + 50 °C. Mobile phases, delivered at 400 µl/min, consisted of H_2_O (eluent A) and MeOH (eluent B), both containing 0.1% (v/v) of HCOOH. The following gradient profile was used: 0–10 min: 2 → 100% eluent B, 10–14.5 min: 100% eluent B, 14.5–14.51 min: 100 → 2% eluent B, and 14.51–16.5 min: 2% eluent B. In the HILIC method, 2 μl of the sample solution was injected onto a column (Acquity UPLC BEH Amide column, 2.1 × 100 mm, 1.7 μm, Waters Corporation, Milford, MA, USA) kept at + 45 °C. Mobile phases, delivered at 600 µl/min, consisted of 50% (v/v, eluent A) and 90% (v/v, eluent B) ACN, both containing 20 mM NH_4_HCO_2_ (pH 3). The following gradient profile was used: 0–2.5 min: 100% eluent B, 2.5–10 min: 100 → 0% eluent B, 10–10.01 min: 0 → 100% eluent B, and 10.01–12.5 min: 100% eluent B.

A Jetstream ESI source, operated in both positive and negative ionization modes, was used under the following conditions: drying gas temperature + 325 °C and a flow of 10 l/min, sheath gas temperature + 350 °C and a flow of 11 l/min, nebulizer pressure 45 psi, capillary voltage 3500 V, nozzle voltage 1000 V, fragmentor voltage 100 V, and skimmer 45 V. N_2_ was used as the instrument gas. For data acquisition, a 2 GHz extended dynamic range mode was utilized in both positive and negative ion modes from *m/z* 50 to 1600. The data were collected in the centroid mode at an acquisition rate of 1.7 spectra/s (599 ms/spectrum) with an abundance threshold of 150. For the automatic data-dependent MS/MS analyses, the precursor isolation width was 1.3 Da, and from every precursor a scan cycle of 4 most abundant ions was selected for fragmentation. These ions were excluded after 2 product ion spectra and released again for fragmentation after a 0.25 min hold. Precursor scan time was based on ion intensity, ending at 25,000 counts or after 300 ms. The product ion scan time was 300 ms, and the collision energies were 10, 20, and 40 V in subsequent runs. The TOF was calibrated on a daily basis and operated at high accuracy (< 2 ppm). Continuous mass axis calibration was performed by monitoring 2 reference ions from an infusion solution throughout the runs. The reference ions were *m/z* 121.050873 and *m/z* 922.009798 in the positive mode and *m/z* 112.985587 and *m/z* 966.000725 in the negative mode, respectively.

### Data Acquisition and Feature Finding in Non-targeted Metabolite Profiling

The 2-pass feature extraction process was used to find molecular features from the data. Initially, the data were deconvoluted into individual peaks with the Agilent MassHunter Profinder B.08.00 software, using the Batch Molecular Feature Extraction algorithm. It took into account all ions exceeding 3000 and 2000 counts with the RP and HILIC methodologies, respectively. Isotope grouping was based on the common organic molecules model. Adduct deconvolution was not utilized. The resulting feature files were subsequently imported into the Mass Profiler Professional 12.6.1 software (Agilent Technologies), which aligned, normalized, visualized, and filtered the features further. During this process, the data were filtered to create a consensus feature list based on a relative frequency threshold, which corresponds to the number of features found in a defined percentage of at least one or more conditions, *i.e.*, C, RA, and pOA, for the various sample replicates. Relative frequency threshold values of 25% in all methodologies and both sample types were used for the first pass data filtering with RP ESI + , RP ESI − , HILIC ESI + , and HILIC ESI − modes. The second pass employed the consensus feature list previously constructed with the Mass Profiler Professional and Profinder’s Batch Targeted Molecular Feature Extraction workflow. The consistency of peak integration was inspected, and a missing feature recovery was performed across all samples during the second pass. The data were then imported for further statistical analyses.

The original data consisted of 21,277 molecular features in 4 separate analytical methodologies (SF: 2546 in HILIC ESI + , 561 in HILIC ESI − , 3926 in RP ESI + , and 1746 in RP ESI − ; IFP: 3228 in HILIC ESI + , 1402 in HILIC ESI − , 4179 in RP ESI + , and 3689 in RP ESI −). After frequency-based filtering, 8125 SF features (2376 HILIC ESI + , 501 HILIC ESI − , 3638 RP ESI + , and 1610 RP ESI − features) and 11,527 IFP features (3005 HILIC ESI + , 1261 HILIC ESI − , 3946 RP ESI + , and 3315 RP ESI − features) remained.

### Data Preprocessing

Data preprocessing consisted of (base-2) logarithmic transformation of signal area values, filtering and imputing missing values, and variance filtering. All preprocessing scripts were implemented with the AWK programming language (GNU Awk *v*4.1.4). In the SF data, 41.0% of 8125 features contained missing values (11.9% of all area values), while in the IFP data, 46.4% of 11,527 features had missing values (13.6% of all area values). Missing values were handled in a 2-phase manner. First, the features with an excessive number of missing values were filtered with the modified 80% rule [20], which saves a feature if any biological group contains at least 80% of non-missing values. The underlying motivation of the modified 80% rule is to be able to detect associations where a molecular feature is present in one group, even if it is absent (presumably below the detection limit) in others. However, if only one group contains a sufficient number of non-missing values, any subsequent testing can only be performed against this group (*i.e.*, one cannot compare distributions in two groups that both contain over 20% missing values).

Next, missing values were imputed with a 3-phase approach that first evaluated the type of missingness, either missing at random/completely at random or missing not at random (MNAR, presumably left-censored measurements), and then imputed both types of missing values separately. For the MNAR imputation, we used a new efficient random tail imputation method, the details of which are described in Additional file 2. Data preprocessing was completed with variance filtering that removed the features, whose variance was at most 1.0. The motivation of variance filtering is to reduce the number of hypotheses by pruning features that are unlikely to differentiate the groups, without risking the control of type I error rate in subsequent *t*-tests [21]. After variance filtering, 2188 SF features and 3576 IFP features remained.

### Univariate Analysis

In the univariate analysis, remaining molecular features were evaluated individually for their ability to separate two groups, *i.e.*, RA *vs.* C, pOA *vs.* C, and pOA *vs.* RA in the SF data and pOA *vs.* RA in the IFP data. The tests were only performed for those pairs where both groups contained less than 20% imputed values (modified 80% rule), which left 6183 tests in the SF data. In the IFP data, there were only two groups, and no further filtering was needed for the 3576 tests.

For each feature and pair of groups, we performed Welch’s *t*-test (unequal variances *t*-test) to obtain raw *p* values (*p_*_*orig*_) and used the Benjamini–Hochberg–Yekutieli method [22] to control the false discovery rate (FDR), yielding FDR-corrected *p* values (*p_*_*FDR*_). The more powerful Benjamini–Hochberg method [23] could not be utilized since there were also negative correlations between features. In addition, the effect size was evaluated with *log-FC* (fold change) [24]:

$$log{\text{-}}FC=avg(log(xi))-avg(log(yi)),$$

where *x*_*i*_ and *y*_*i*_ are signal area measurements in two groups. Since log-area reflects the concentration of a compound fragment, *p* values and *log-FC* can be used to detect interesting compounds (possible metabolites) that have significantly higher abundance in one group than in another. Summaries of *log-FC* and *t*-test analysis were presented visually as volcano plots. Since *log-FC* and *t*-statistics are sensitive to outliers and the accuracy of imputed values, we also determined minimum margin size between distributions for detecting well separating features (Additional file 2).

After univariate analysis, the most significant features having *p_*_*orig*_ ≤ 0.01 and *|log-FC|*≥ 1 were selected for identification. During identification, further filtering was performed by pruning features with low quality, missing MS/MS data, or duplicate information (*i.e.*, adducts, fragments, or dimers).

Univariate analysis was performed with the MATLAB *v*9.6 R2019a using the Statistics and Machine Learning Toolbox (MathWorks, Natick, MA, USA), and volcano plots were produced with Python using the Matplotlib library [25].

### Multivariate Analyses

The multivariate analyses consisted of principal component analysis (PCA) and linear discriminant analysis (LDA). They were first performed with all features and again after identified drugs had been removed from the filtered data sets. In PCA, we calculated principal components and plotted the data along the first 3 principal components to detect if linear combinations of features could separate groups. In LDA, the goal was to test how well the groups could be separated by linear discriminant functions of selected features. LDA is a simple classifier that often performs better than more complex models when the number of samples is small, but the model (its large covariance matrix) cannot be estimated reliably, when the number of features is excessive.

Two solutions to improve the model stability and performance are feature selection and the use of regularization. Extensive feature selection was already performed during filtering, but the numbers of features were still much larger than sample sizes (185 features in the SF data and 75 features in the IFP data after removing drugs). Therefore, we tested 2 additional feature selection strategies: (1) features having *p_*_*FDR*_ ≤ 0.05 and *|log-FC|*≥ 2 and (2) features having *p_*_*FDR*_ ≤ 0.5 and *|log-FC|*≥ 1. For regularization, we used the shrinkage regularization method by Ledoit and Wolf [26], where the idea is to shrink individual covariance estimates towards average covariance.

The classification performance was evaluated with leave-one-out cross-validation by calculating overall accuracy (proportion of correct classifications), precision, and sensitivity for each class. The cross-validation results of the best feature selection strategy 2 (*p_*_*FDR*_ ≤ 0.5 and *|log-FC|*≥ 1) as well as the corresponding scatter-plots along discriminants (2 discriminants for the SF and 1 for the IFP data) are presented in the “RESULTS” section. PCA and LDA were implemented with Python using the scikit-learn library [27], and the visualizations were produced with Python using the Matplotlib library [25].

### Identification of Metabolites

Molecular features with significant differences between study groups were subjected to identification of metabolites based on accurate mass and isotope information, *i.e.*, ratios, abundances, spacing, and product ion spectra (MS/MS) by spectral matching to published databases as follows: Human Metabolome Database (https://hmdb.ca/), ChemSpider (https://chemspider.com), mzCloud (https://mzcloud.org), LIPID MAPS (https://lipidmaps.org), MS-DIAL (http://prime.psc.riken.jp/compms/msdial/main.html), and MassBank (https://massbank.eu/MassBank/). When available, the fragmentation was verified with a commercial standard. During the first step, molecular features were identified by accurate mass and isotopic pattern matching with the METLIN database (https://metlin.scripps.edu) by using Agilent’s Identification Browser software. The results were sorted and an assessment of retention time and a single putative annotation with a matching elemental formula was selected. This annotated molecular feature was further compared to other databases. Data-dependent product ion spectra were acquired at 3 collision energies (10, 20, and 40 eV), and the MS/MS spectra of statistically significant molecular features were compared and matched to a library of standard spectra in Agilent’s MassHunter METLIN and in the above-mentioned databases. The confidence level of metabolite identification was established according to the Metabolomics Standards Initiative reporting standard [28]. Only the metabolites that were identified at levels 1–2 are discussed below, but all metabolites of levels 1–4 are reported in Additional files 3–4.

## RESULTS

### Demographic Characteristics

The baseline characteristics of the patients are summarized in Table 1. There were no significant differences in the sex ratios or average body masses between diagnoses. The pOA and RA patients were significantly older than the C group, and the pOA patients had higher average BMIs compared to the RA group.

### Univariate Analyses of Metabolites

**Fig. 1:**
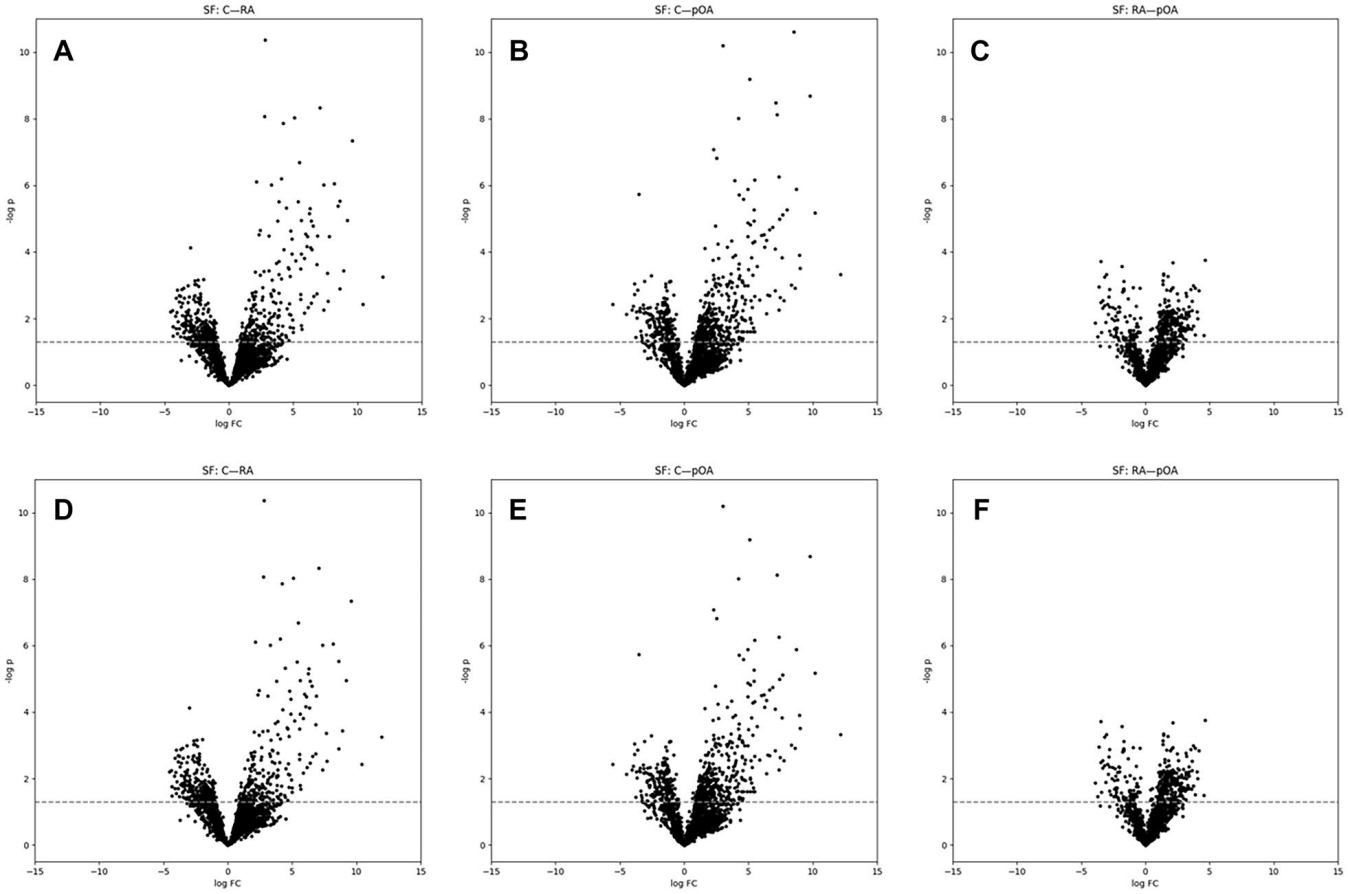
Volcano plots from the synovial fluid (SF) data. Volcano plots summarize results of univariate testing (RA *vs.* C, pOA *vs.* C, and pOA *vs.* RA) along *log-FC* (*x*-axis) and −*log*(*p*) (*y*-axis) from the Welch’s *t*-test using all features (**A**–**C**) and features without identified drugs (**D**–**F**). Negative logarithm of the nominal level *p* = 0.05 is marked with a dash line. C, control; RA, rheumatoid arthritis; pOA, primary osteoarthritis.

**Fig. 2:**
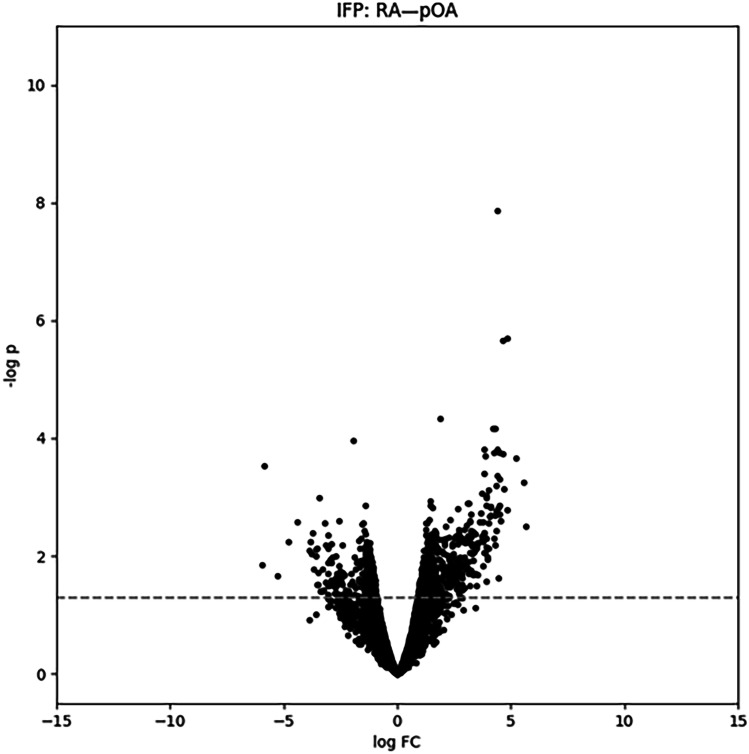
Volcano plot from the infrapatellar fat pad (IFP) data. Volcano plot summarizes results of univariate testing (pOA *vs.* RA) along *log-FC* (*x*-axis) and − *log*(*p*) (*y*-axis) from the Welch’s *t*-test. All features are presented since no drugs were identified among the significant features in the IFP data. Negative logarithm of the nominal level *p* = 0.05 is marked with a dash line. RA, rheumatoid arthritis; pOA, primary osteoarthritis.

Volcano plots show that multiple molecular features could clearly separate both RA and pOA SFs from C SF (Fig. 1A–B, D–E), but differences between RA and pOA were more modest in both SF and IFP (Fig. 1C, F and 2). The fact that the volcano plots for pOA *vs.* C and RA *vs.* C were asymmetrical (skewed to right) means that many features occurred at higher abundances in the pOA and RA groups, even after removal of identified drugs. After all filtering (*p_*_*orig*_ ≤ 0.01, *|log-FC|*≥ 1, and further pruning during identification), 170 SF features and 73 IFP features expressed significant differences between study groups. Among these, 19 SF features could separate pOA, RA, or both from C with *p_*_*FDR*_ ≤ 0.05.

In SF, both pOA and RA groups showed higher levels of particular therapeutic agents (omeprazole, gabapentin, and tranexamic acid) than the C patients, and the RA patients had higher presence of sulfasalazine. In IFP, no drug-derived substances could be identified among features that showed statistical differences. In addition, 16 and 13 metabolites with significant differences between diagnoses were identified in SF and IFP, respectively. These mostly belonged to the pathways of androgen, bile acid, glycero-PL, AA, histamine, biotin, and purine metabolism (Table 2; Additional files 3–4).

**Table 2:** Identified Metabolites Significantly Associated with Primary Osteoarthritis (pOA) or Rheumatoid Arthritis (RA) Synovial Fluid (SF) or Infrapatellar Fat Pad (IFP)

**pOA SF** ***vs.*** **control SF**	**RA SF** ***vs.*** **control SF**	**RA SF** ***vs.*** **pOA SF**	**RA IFP** ***vs.*** **pOA IFP**
Phosphatidylethanolamine (22:6, 16:0) ↑	Testosterone sulfate ↓	Testosterone sulfate ↓	Testosterone sulfate ↓
Lysophosphatidylcholine (16:0) ↑	Androsterone sulfate ↓	Cholic acid ↓	Androsterone sulfate ↓
Palmitoleamide ↓	Lysophosphatidylcholine (16:0) ↑	Chenodeoxycholic acid ↓	Cholest-4-en-26-oic acid, 7*α*-hydroxy-3-oxo ↓
Oleamide ↓	Deoxyguanosine ↑	Lysophosphatidylcholine (16:0) ↓	Phosphatidylcholine (16:0, 16:0) ↑
Oleoyl ethylamide ↓	Gluconic acid ↑	Lysophosphatidylcholine (18:2) ↓	Lysophosphatidylcholine (18:0) ↓
Linoleamide ↓		Lysophosphatidylcholine (18:3) ↓	L-Arginine ↓
CMPF ↑			Proline ↓
Deoxyguanosine ↑			Glutamic acid ↓
7-Keto-8-aminopelargonic acid ↑			Aspartic acid ↓
Gluconic acid ↑			L-Pipecolic acid ↓
			Histamine ↓
			4-Imidazoleacetic acid ↓
			Guanidineacetic acid ↓

*CMPF* 3-carboxy-4-methyl-5-propyl-2-furanpropanoic acid, ↑ significant elevation in levels, ↓ significant decrease in levels (Welch’s *t*-test, *p* < 0.05)

### Multivariate Analyses (LDA, PCA) of Metabolites

**Fig. 3:**
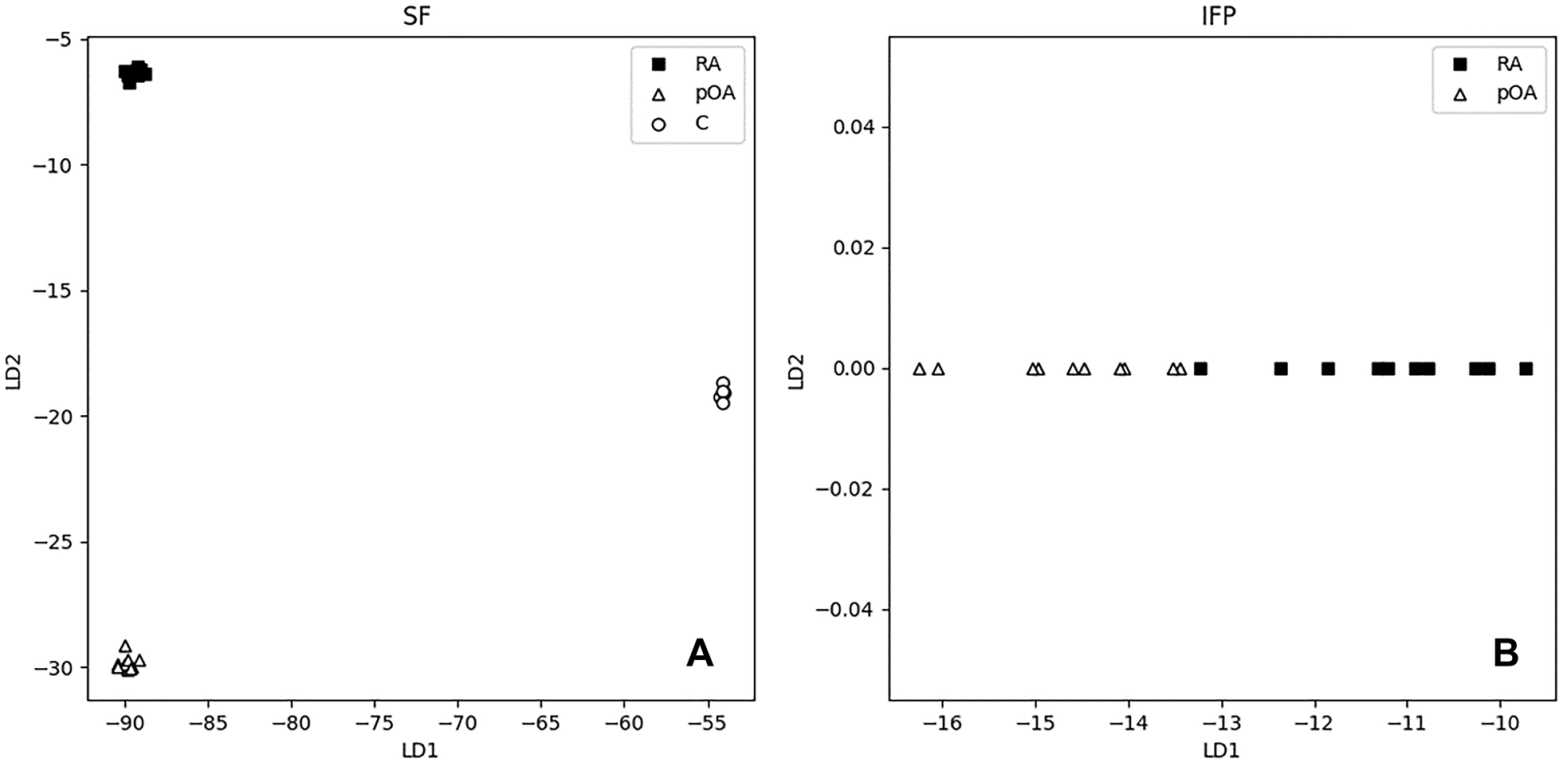
Linear discriminant analysis (LDA) scatter-plots of the synovial fluid (SF) and infrapatellar fat pad (IFP) data. LDA scatter-plots of the filtered SF data (**A**) and IFP data (**B**) along the linear discriminants (LDs). In the SF data, there were three classes and, thus, two LDs (*x*- and *y*-axes) while in the IFP data, there were only two classes and, thus, one LD (*x*-axis). C, control; RA, rheumatoid arthritis; pOA, primary osteoarthritis.

In the SF data, the most accurate LDA models were obtained with filtering |*log-FC*|≥ 1.0 and either *p_*_*FDR*_ ≤ 0.5 or *p_*_*orig*_ ≤ 0.01. There were no misclassifications in the cross-validation and, thus, the overall accuracy as well as precision and sensitivity in all three classes were 100%. Deoxyguanosine, lysophosphatidylcholine LPC(16:0), 3-carboxy-4-methyl-5-propyl-2-furanpropanoic acid (CMPF), LPC(18:3), testosterone sulfate, cholic acid, and gluconic acid were among the most important identified molecules that discriminated the diagnoses. Figure 3A presents a scatter-plot of the entire data along the 2 discriminants using the stricter filtering (*p_*_*FDR*_ ≤ 0.5), where the model was constructed from 69 features.

In the IFP data, filtering with |*log-FC*|≥ 1.0 and *p_*_*FDR*_ ≤ 0.5 (4 features) yielded the best compromise in terms of cross-validation and class separation in the entire data. In cross-validation, the overall accuracy was 90%, precision 100% (in RA) or 83% (pOA), and sensitivity 100% (RA) or 80% (pOA), respectively. 4-Imidazoleacetic acid was among the most significant contributors to the model. In the entire data, the only discriminant separated the classes perfectly (Fig. 3B). With stricter filtering |l*og-FC*|≥ 2 and *p_*_*FDR*_ ≤ 0.05 (only 1 feature), the cross-validation accuracy was better (95%), but the class separation in the entire data was worse. The loosest filtering with *p_*_*orig*_ ≤ 0.01 (75 features) produced the worst accuracy (85%), which suggests that the model suffered from overfitting.

**Fig. 4:**
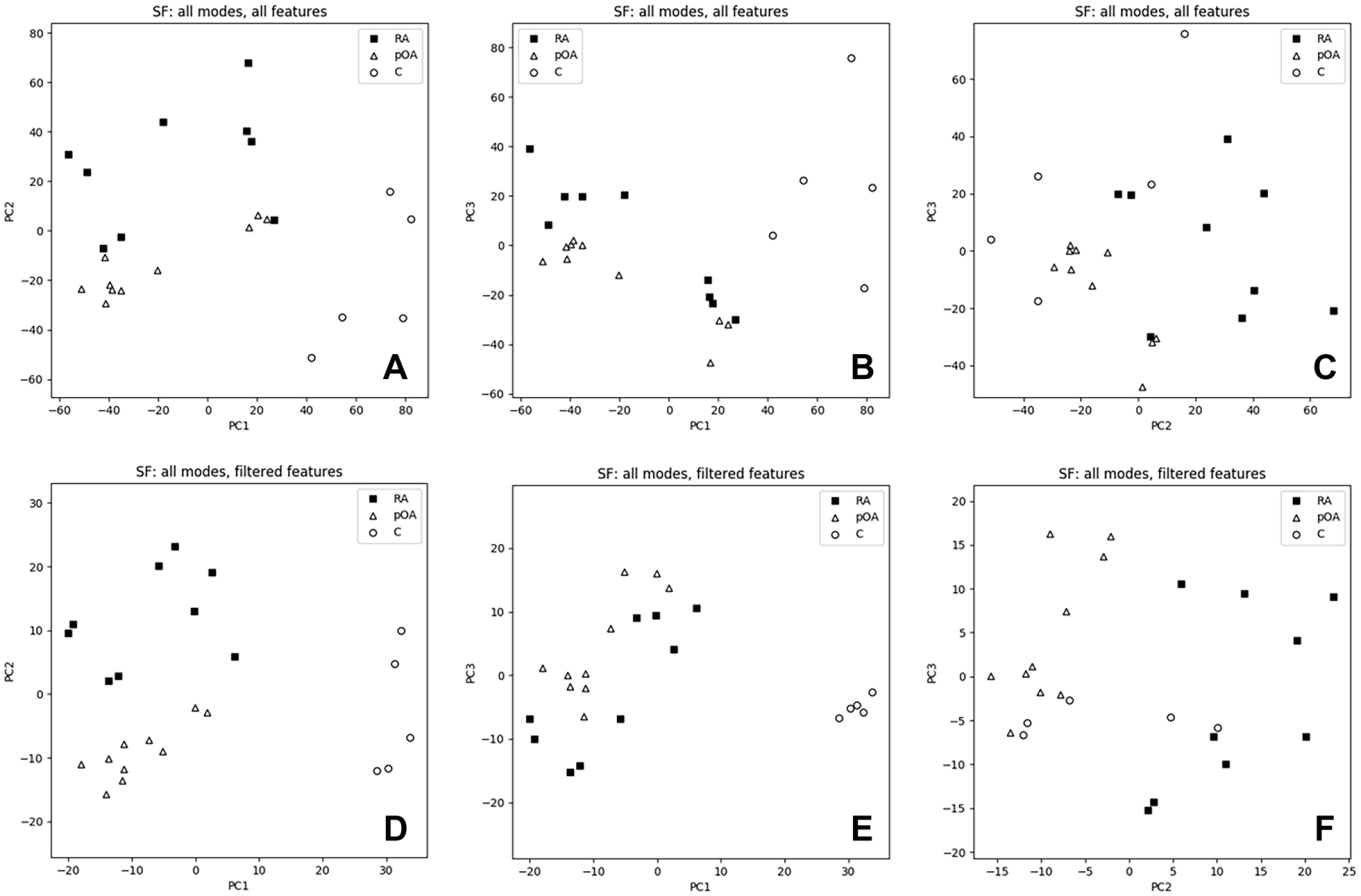
Principal component analysis (PCA) scatter-plots of the synovial fluid (SF) data. PCA scatter-plots of the SF data along the first three principal components (PCs): all features (**A**–**C**) and filtered features (**D**–**F**). C, control; RA, rheumatoid arthritis; pOA, primary osteoarthritis.

**Fig. 5:**
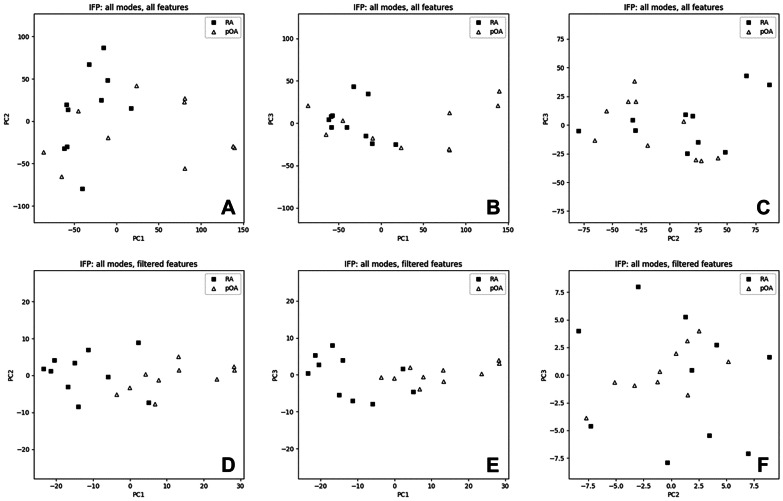
Principal component analysis (PCA) scatter-plots of the infrapatellar fat pad (IFP) data. PCA scatter-plots of the IFP data along the first three principal components (PCs): all features (**A**–**C**) and filtered features (**D**–**F**). RA, rheumatoid arthritis; pOA, primary osteoarthritis.

Results of the PCA are shown in Figs. 4 and 5. In the SF data, the first 2 principal components could clearly separate C from pOA and RA already before filtering (Fig. 4A), and all 3 classes were well separated after filtering (Fig. 4D). In the IFP data, the class separation was less clear but, after filtering, the first 2 principal components could almost separate RA and pOA (Fig. 5D).

## DISCUSSION

### General Comments and Drug-Derived Molecules

Based on previous literature, it is known that the SF metabolome can be affected by degenerative joint diseases. However, while circulation, synovial membrane, and immune cells are recognized sources of SF molecules, the potential role of IFP as an effector of knee SF composition remains significantly less investigated. Here, we conducted a pilot study about the effects of pOA and RA on not only the SF metabolomics but also on the adjacent fat pad to examine, if there would be parallel changes in these tissues that would be relevant to joint diseases, to search for promising biomarkers that could be applied to studying early-stage OA and RA, and to assess potential metabolites that would offer useful targets for therapeutic manipulation. Below, we concentrate on the statistically and biologically most significant differences between the diagnoses regarding the identified molecules among filtered features (*p_*_*orig*_ ≤ 0.01 and *|log-FC|*≥ 1).

In SF, a few clearly drug-derived molecules–omeprazole, gabapentin, and tranexamic acid–were present in excess in pOA and RA samples, as expected. Omeprazole is a proton-pump inhibitor that is widely utilized to counteract the gastrointestinal side-effects of non-steroidal anti-inflammatory drugs [29]. Gabapentin alleviates (neuralgic) pain, and it is not necessarily prescribed for knee pOA or RA. As degenerative diseases also affect other skeletal structures, the use of this medication would be understandable considering the often-concomitant spondyloarthritis with nerve root symptoms. Tranexamic acid is commonly used in orthopedic surgery to control excessive bleeding [29] and, not surprisingly, was present in large-scale arthroplasty in significantly higher levels than in controls that had undergone smaller procedures. Finally, sulfasalazine as an anti-rheumatic medical agent [29] was present at higher levels in RA but not in pOA SF when compared to C SF. While the appearance of these therapeutic agents was expected, their presence increased the overall validity of the metabolomic analysis, and we suggest that reporting common medical substances when present would provide a good standard for the reliability of the other results, too. In this study, the multivariate statistical analyses were first performed with these identified therapeutic agents but also after their removal from the features.

### Androgen and Bile Acid Pathways

RA was associated with altered androgen and bile acid metabolic pathways in both SF and IFP. It was also previously established that androgen metabolism would be affected by RA, observed as decreased levels of androgens and dehydroepiandrosterone sulfate in both genders [30]. In concert with this, the testosterone sulfate and androsterone sulfate levels were lower in RA IFP compared to pOA IFP, and in RA SF in relation to C SF. In addition, testosterone sulfate was reduced in RA SF *vs.* pOA SF. As a metabolite of testosterone, its sulfate is a weak androgen, while androsterone sulfate is a major androgen metabolite in urine [31, 32]. In the body, the steroid sulfatase enzyme catalyzes the hydrolysis of sulfate ester bonds from a wide array of substrates [33]. In steroid-dependent cancers, steroid sulfatase inhibitors have been proposed as potentially useful therapeutic agents, while in inflammatory diseases their significance is less well established. However, Mueller et al. [33] implied that steroid sulfatase dysregulation could also play a role in inflammatory phenomena. Together with previous data [30], our results suggest that further research into androgen metabolism and, specifically, into modifying steroid sulfatase activity could provide a promising target for therapeutic intervention in joint diseases.

Regarding bile acids and their biosynthesis, the RA patients showed lower levels of particular compounds compared to the pOA patients. In SF, there were decreases in cholic acid and chenodeoxycholic acid, while in IFP the levels of cholest-4-en-26-oic acid, 7*α*-hydroxy-3-oxo were lower in RA than in pOA. These metabolites belong to the pathways of bile acid biosynthesis [34]. Abnormalities of bile acid metabolism have not been intensively studied in RA patients but, in a previous report, their cholic acid, deoxycholic acid, and total bile acid pools, as well as the synthesis rate of cholic acid were suggested to be reduced [35]. In gout, another type of inflammatory arthritis characterized by uric acid crystals, serum markers of primary bile acid biosynthesis can also be decreased [36]. Bile acid metabolites could offer an additional tool to either monitoring of disease activity (decreased levels) or interventions (enhancing bile acid biosynthesis) in RA. In fact, it was previously observed that one of the conjugated bile acids, taurochenodeoxycholic acid, induced anti-arthritis activity in rats [37]. In humans, peroral therapy with chenodeoxycholic acid was investigated as early as in the 1970s with an initial worsening of symptoms followed by remission after approximately 6 weeks [38], both studies yielding further support to the possibility of bile acids becoming useful tools in translational studies on RA.

### Lipid Metabolites

Several RA- and/or pOA-related alterations were observed in the levels of particular glycero-PLs, mostly LPCs, in SF and IFP. The unsaturation and chain length of PL fatty acyl chains can hypothetically affect the lubricating properties of SF and consequently the friction between the surfaces of articular cartilage [17]. The levels of LPC(18:0), LPC(18:2), and LPC(18:3) showed lower values in RA *vs.* pOA in SF or IFP, and there was an increase in the levels of LPC(16:0) in both arthropathies compared to C SF. These results displayed some resemblance to a previous study by Kosinska et al. [17]. In general, LPCs are major components of oxidized low-density lipoproteins, they can have pro-inflammatory properties in vitro, and may play a role in the pathogenesis of, for instance, atherosclerosis [39]. Accordingly, an increased plasma ratio of LPC to phosphatidylcholine (PC) was associated with advanced knee OA [40], and the plasma PC/LPC ratio increased in RA patients receiving anti-inflammatory therapy [41].

Regarding the other findings of lipidology, the increase in CMPF levels in pOA SF when compared to C SF could be a sign of self-medication with fish-based oils rich in long-chain n-3 PUFAs [42]. Furthermore, the pOA patients exhibited decreased levels of palmitoleamide, linoleamide, oleamide, and oleoyl ethylamide *vs.* C SF. Little is known about the significance of FA amides in joint diseases, but oleamide has been shown to be anti-inflammatory in vitro and in an in vivo rodent model [43]. Its decrease could, thus, be associated with inflammation, but it remains to be determined, why this effect did not reach significance in RA SF. Oleamide is also known to accumulate in cerebrospinal fluid of sleep-deprived animals and is able to induce sleep [44]. It can also derive from plastic products, but this does not readily explain the observed differences in its levels between study groups. Among the lipid manifestations of joint disease metabolomics, LPC(16:0) and oleamide could offer potential targets for biomonitoring disease activity. Oleamide could also offer a therapeutic tool to reduce inflammation as well as to provide possible amelioration to joint pain-related sleep disorders.

### Amino Acids and Their Derivatives

We observed significant changes in some metabolites only in IFP but not in SF. In these, alterations were documented in the levels of several AAs, as the L-arginine, proline, aspartic acid, glutamic acid, and L-pipecolic acid levels of the pOA patients were higher than those of the RA group. In addition, guanidineacetic acid increased in pOA IFPs compared to RA IFPs. Once again, previous literature about this subject is somewhat controversial, and some studies show similarity to ours with reduced AA values in RA SF or serum [14, 45–47]. Proline, glutamic acid, aspartic acid, and arginine are known to be important constituents of collagen [48], and their higher levels in pOA IFPs could hypothetically be related to OA-induced fibrosis [8].

L-Pipecolic acid is an interesting metabolite of lysine catabolism that offers enhanced systemic resistance to bacterial infections in experimental organisms [49]. This can be associated with its propensity to increase the levels of free radicals, nitric oxide, and reactive oxygen species [50] but, hitherto, these effects have mostly been demonstrated in plant eukaryotes. In vitro, L-pipecolic acid may cause oxidative stress in cerebral cortex and could, thus, be regarded deleterious [51]. On the other hand, it can increase the rate of protein synthesis [52] and, for this reason, its reduction could be theoretically useful considering the overgrowth of RA synovium as pannus formation. According to Teitsma et al. [46], L-pipecolic acid levels were higher in RA patients who were in drug-free remission after tocilizumab treatment than in patients who never achieved a drug-free status. This fits our results with higher L-pipecolic acid levels in the pOA than in the RA group, both including symptomatic patients only. Similar to the situation of higher L-pipecolic acid levels in RA patients in less inflammatory remission, our pOA patients probably had less systemic inflammation than the RA patients [9]. Thus, L-pipecolic acid has some potential to become a measure of disease remission and resolution of inflammation, at least regarding RA.

A clear difference was also observed for IFP histamine and its metabolite 4-imidazoleacetic acid with higher levels in the pOA than RA patients. This may be related to, for instance, drug interactions, as sulfasalazine is known to inhibit histamine release [53]. Low histamine levels were also previously observed in the circulation and SF of RA patients [54]. However, while Adlesic et al. [54] noted that histamine would have no effects on inflammation in vitro or in an in vivo rodent model, the present observation of higher histamine levels in pOA merits further investigation, as it is a well-known inflammatory agent that can be easily manipulated with existing remedies. In fact, Shirinsky and Shirinsky [55] suggested that the use of H_1_-antihistamines would be associated with a reduced prevalence of knee OA. It was also observed by Tetlow and Woolley [56, 57] that histamine may induce the production of cartilage-degrading proteinases and the formation of chondrocyte clusters associated with OA, which yields more support from our results. While the issue remains somewhat controversial, our data join the increasing amount of literature suggesting that a closer and novel look into histamine-targeted therapies, especially regarding OA, is warranted.

### Other Significant Molecules in SF

In SF only, there was a marked increase in the deoxy-guanosine levels by both pOA and RA compared to C. Despite different etiologies, both arthritic diseases could share a common pathological pathway in this respect. This finding fits well with the inflammatory nature of OA and RA, as it has been established that increases in 8-hydroxy-2’-deoxyguanosine indicate oxidative DNA damage and inflammation [58]. Thus, while there exist different deoxyguanosines and the precise molecular formula of the one measured in this study could unfortunately not be determined, we can conclude that oxidatively damaged DNA adducts could be provisionally good candidates for SF biomarkers of inflammatory joint diseases, while they do not seem to be as promising as agents for therapeutic interventions.

Of the other SF molecules with significant differences between diagnoses, 7-keto-8-aminopelargonic acid (KAPA) is an intermediate in biotin (vitamin B7) biosynthesis by intestinal bacteria [59, 60], and it was higher in the pOA than in the C knees. Recently, biotin has been implicated in various inflammatory phenomena [61, 62], and this makes the KAPA results intriguing. At this stage, however, the inflammatory effects of this biotin metabolite remain elusive and further studies are required, especially regarding joint diseases. Yet another interesting molecule with elevated levels in both pOA and RA SFs is gluconic acid, which is most probably derived from the patients’ diet, as it occurs naturally in, for instance, fruits and honey [63]. Its salt, gluconate, is being investigated for its potential use in cancer therapies. At the moment, the practical applications of gluconic acid for joint diseases remain to be determined, but still both gluconic acid and the biotin biosynthesis pathway can provide starting points to gather more knowledge on the metabolic alterations that participate in the pathogeneses of OA and RA.

### Potential Clinical Implications

The main debilitating symptom of OA and RA is pain. To offer effective treatment options to patients, it would be useful to assess, which metabolites and metabolic pathways would either be the most responsible for the perceived pain or could offer potential remedies. How the metabolites listed above correlate to pain in joint diseases has not been intensely researched, but for other conditions there are available data. Regarding pain in general, epiandrosterone sulfate correlated inversely with the risk of chronic widespread musculoskeletal pain [64]. This suggests that the observed higher levels of androgen sulfates in the present study would probably not be contributing factors to the pathogenesis of pOA but might become a useful tool in the study and treatment of symptomatic pain. Levels of individual AAs in cerebrospinal fluid have also been noted to associate with the intensity of pain [65]. Arginine and glutamine are examples of AAs that can become deficient in diverse medical conditions, such as trauma, infection, and intestinal diseases [66] and may, thus, be related to inflammatory responses. The observed lower L-arginine values in the more inflammatory RA compared to pOA could fit this pattern and offer a promising therapeutic target. AAs could prove to be metabolites of several beneficial interventions, as they can modulate immune responses and, for instance, arginine is required for normal proliferation and maturation of T-cells, and it is involved in the macrophage class transition from M1 to M2, driving inflammation and resolution [66]. Bile acids [67] and pro-inflammatory LPCs [68] represent other metabolites that can be related to chronic pain and should be studied in more detail regarding joint diseases.

### Study Limitations

The present study had some limitations to be acknowledged. Several statistically significant features could not be identified with reliable accuracy down to the molecular level, due to which they had to be left out of the discussion. The group size in this pilot study was also relatively small. As sampling for IFP would have affected the control patients’ surgery procedures and caused additional invasive manipulation with ethical permits difficult to obtain, IFP samples could only be harvested from the pOA and RA patients. In these groups, the sampling of IFP did not significantly affect the course of the default operation, as during arthroplasty lots of tissue is removed. However, this narrows the scope of the conclusions that could be drawn from the IFP results.

Finally, it must be acknowledged that due to the inevitable natural progress of OA and RA, knee replacement surgery patients tend to be significantly older than knee trauma patients, who are usually of the younger generation, physically more active, and prone to accidents in sports causing joint damage. These issues are recurring confounding factors in joint disease research, and age-related dissimilarities between study groups could, thus, have masked or augmented some of the observed differences when pOA and RA SFs were compared to C SF. This could be especially relevant regarding the androgen and bile acid metabolites that generally show decreasing levels with advancing age [69, 70], but as these compounds mostly displayed differences between the pOA and RA patients that had similar ages, they could be reliably used to assess the differences between these two joint diseases. In contrast, the deoxyguanosine levels tend to increase with aging [71], and as they showed significantly higher values in pOA and RA SFs compared to C SF, age-related effects cannot be excluded in this case. In addition, there was a significant difference in BMIs between the pOA and RA patients, and the potential influence of adiposity on their divergent metabolomic profiles should be recognized.

## CONCLUSIONS

There was parallel downregulation of androgen and bile acid metabolism by RA in both SF and the adjacent IFP. L-Pipecolic acid, deoxyguanosine, LPC(16:0), and oleamide could be potential biomarkers for oxidative stress and inflammation in end-stage pOA and/or RA. Further studies are required to find out if there could be associations of these metabolites with early-stage joint diseases or with pain parameters. Testosterone/androsterone sulfates, bile acids, histamine, L-arginine, and oleamide are among metabolites that could be useful targets for therapeutic manipulation. In addition, some interesting but scarcely investigated pathways, especially biotin metabolism, offer attractive future research targets.

## Supplementary Information

Below is the link to the electronic supplementary material.

Supplementary file1 (DOCX 20 KB)

Supplementary file2 (DOCX 16 KB)

Supplementary file3 (XLSX 51 KB)

Supplementary file4 (XLSX 49 KB)

## Data Availability

All relevant data generated or analyzed during this study are included in this published article and its supplements.

## Abbreviations

AA: Amino acid
ANOVA: Analysis of variance
BMI: Body mass index
C: Control
CMPF: 3-Carboxy-4-methyl-5-propyl-2-furanpropanoic acid
ESI: Electrospray ionization
FA: Fatty acid
FC: Fold change
FDR: False discovery rate
HILIC: Hydrophilic interaction chromatography
IFP: Infrapatellar fat pad
KAPA: 7-Keto-8-aminopelargonic acid
LDA: Linear discriminant analysis
LPC: Lysophosphatidylcholine
MNAR: Missing not at random
OA: Osteoarthritis
PC: Phosphatidylcholine
PCA: Principal component analysis
PL: Phospholipid
pOA: Primary osteoarthritis
PUFA: Polyunsaturated fatty acid
RA: Rheumatoid arthritis
RP: Reversed phase
SF: Synovial fluid
UHPLC-qTOF-MS: Ultra-high-performance liquid chromatography quadrupole-time-of-flight mass spectrometry
