# Supplementary material for: Metabolomics of Synovial Fluid and Infrapatellar Fat Pad in Patients with Osteoarthritis or Rheumatoid Arthritis
Source: Inflammation. 2022 Jan 18;45(3):1101–17. doi: 10.1007/s10753-021-01604-x (PMC9095531; doi:10.1007/s10753-021-01604-x)
Supplement: Supplementary file 1 — Supplementary file1 (DOCX 20 KB) [file 10753_2021_1604_MOESM1_ESM.docx]

**Additional file 1** Diagnostic data of the sampled knee surgery patients

| ID | Group | Gender | Operation | Operative diagnosis |
| --- | --- | --- | --- | --- |
| 01 | Control | F | Arthroscopy, ACL reconstruction | M23.5, M23.2 |
| 02 | Control | F | Diagnostic arthroscopy | M23.5 |
| 03 | Control | F | Arthroscopy, debridement | M25.5 |
| 04 | Control | M | Arthroscopy, partial meniscal resection | M23.2 |
| 05 | Control | M | Arthroscopy, ACL reconstruction | S83.5 |
| 06 | RA | F | Total knee replacement | M17.5 (secondary) |
| 07 | RA | F | Total knee replacement | M17.5 (secondary) |
| 08 | RA | F | Total knee replacement | M17.5 (secondary) |
| 09 | RA | F | Total knee replacement | M17.5 (secondary) |
| 10 | RA | M | Total knee replacement | M17.5 (secondary) |
| 11 | RA | F | Total knee replacement | M17.4 (other secondary) |
| 12 | RA | F | Total knee replacement | M17.4 (other secondary) |
| 13 | RA | M | Total knee replacement | M17.5 (secondary) |
| 14 | RA | M | Total knee replacement | M17.5 (secondary) |
| 15 | RA | F | Total knee replacement | M17.4 (other secondary) |
| 16 | pOA | M | Total knee replacement | M17.1 (primary) |
| 17 | pOA | M | Total knee replacement | M17.1 (primary) |
| 18 | pOA | F | Total knee replacement | M17.1 (primary) |
| 19 | pOA | F | Total knee replacement | M17.1 (primary) |
| 20 | pOA | F | Total knee replacement | M17.1 (primary) |
| 21 | pOA | F | Total knee replacement | M17.1 (primary) |
| 22 | pOA | F | Total knee replacement | M17.1 (primary) |
| 23 | pOA | F | Total knee replacement | M17.1 (primary) |
| 24 | pOA | F | Total knee replacement | M17.1 (primary) |
| 25 | pOA | F | Total knee replacement | M17.1 (primary) |

F = female, M = male, RA = rheumatoid arthritis, pOA = primary osteoarthritis, ACL = anterior cruciate ligament
