# Supplementary material for: Metabolomics of Synovial Fluid and Infrapatellar Fat Pad in Patients with Osteoarthritis or Rheumatoid Arthritis
Source: Inflammation. 2022 Jan 18;45(3):1101–17. doi: 10.1007/s10753-021-01604-x (PMC9095531; doi:10.1007/s10753-021-01604-x)
Supplement: Supplementary file 2 — Supplementary file2 (DOCX 16 KB) [file 10753_2021_1604_MOESM2_ESM.docx]

**Additional file 2.** Extended methodology

**Missing value imputation**

The new method was designed with the specific aim to save interesting patterns of left-censored values, where most or all values in one group were below the detection limit. The other criteria were efficiency (ability to scale up to thousands of features) and robustness, given the small sample sizes and non-normally distributed features.

The imputation was implemented in 3 phases. First, the type of missingness was determined for each feature. The goal was to detect if values were missing at random/completely at random (MAR/MCAR) or not at random (MNAR), which was interpreted as left-censored data (measurements below the detection limit). This was done by evaluating the expected numbers of missing values per feature in total and in each group separately and by evaluating significant positive deviation (p ≤ 0.10) from the expectation by binomial probability. This led to the following heuristic rules: in synovial fluid, if at most 2 values were missing from a feature and of them at most 1 was in group C (only 5 samples), the type was MNAR. In infrapatellar fat pad, if at most 3 values were missing from the feature and at most 2 values from one group, the type was MNAR. Since all MNAR type missing values are not necessarily left-censored, the decisions were later smoothed by imputing smaller values to features that were most likely to be left-censored, demonstrated by small magnitudes in observed values.

Second, the MAR/MCAR type missing values were imputed with random imputation from a uniform distribution, where the missing value is substituted with a random value from the range of observed values for the given feature. In principle, any efficient MAR/MCAR imputation method could have been used at this step, but one cannot assume normality or even unimodality of individual feature distributions, and the mean and median imputation should be avoided due their underestimation of variance and consequent errors in univariate testing.

Third, the MNAR type missing values were imputed with a new, robust, and efficient random tail imputation that constructs a distribution for the missing left tail and imputes values according to it. The method does not require a normal distribution, but only a unimodal, approximately symmetrical distribution, which is more realistic and allows efficient implementation. The main ideas were as follows: 1. The frequency histogram of observed values was constructed, its peak and truncation point were determined, and a target distribution for the left tail was constructed by mirroring the right tail. If needed, the target distribution was adjusted to make it monotonic. 2. The existing and target distributions of the left tail were compared and, for each bin, the number of extra values required was determined based on the comparison and the number of missing values. 3. The overall frequency density function was presented as piecewise linear function (one function per bin) to smooth the histogram, and the corresponding cumulative functions and their inverses were determined. 4. Missing value positions in data were shuffled into a random order and divided into two groups based on the magnitudes of observed values in their features: larger and smaller values (using the threshold 12). 5. For each bin, beginning from the truncation point and proceeding to the lower end, the needed number of new values was generated randomly according to the inverse of the cumulative frequency function. The generated values were inserted to missing value positions in the determined random order, beginning from those requiring larger values.

**Measurement of minimum margin size**

Minimum margin size (*msize*) is defined as

*msize = max{min(x_i_) − max(y_i_), min(y_i_) − max(x_i_)},*

where *x_i_* and *y_i_* are signal area measurements in two groups. If *msize* is positive, the log-area distributions in two groups are completely separated, and the feature can separate groups perfectly. This measure is more reliable than *log-FC* and t-statistic because outliers and inaccurate imputations can at most underestimate it, but never overestimate.
